# Characterization of a Drosophila Alzheimer's Disease Model: Pharmacological Rescue of Cognitive Defects

**DOI:** 10.1371/journal.pone.0020799

**Published:** 2011-06-06

**Authors:** Ranjita Chakraborty, Vidya Vepuri, Siddhita D. Mhatre, Brie E. Paddock, Sean Miller, Sarah J. Michelson, Radha Delvadia, Arkit Desai, Marianna Vinokur, David J. Melicharek, Suruchi Utreja, Preeti Khandelwal, Sara Ansaloni, Lee E. Goldstein, Robert D. Moir, Jeremy C. Lee, Loni P. Tabb, Aleister J. Saunders, Daniel R. Marenda

**Affiliations:** 1 Department of Biology, Drexel University, Philadelphia, Pennsylvania, United States of America; 2 Department of Biological Sciences, University of the Sciences in Philadelphia, Philadelphia, Pennsylvania, United States of America; 3 Department of Psychiatry, Boston University, Boston, Massachusetts, United States of America; 4 Genetics and Aging Research Unit, MIND, Massachusetts General Hospital, Harvard Medical School, Boston, Massachusetts, United States of America; 5 Department of Molecular, Cell, and Developmental Biology, University of California Santa Cruz, Santa Cruz, California, United States of America; 6 Department of Epidemiology and Biostatistics, Drexel University, Philadelphia, Pennsylvania, United States of America; 7 Department of Biochemistry and Molecular Biology, Drexel University College of Medicine, Philadelphia, Pennsylvania, United States of America; 8 Department of Neurobiology and Anatomy, Drexel University College of Medicine, Philadelphia, Pennsylvania, United States of America; Brigham and Women's Hospital, Harvard Medical School, United States of America

## Abstract

Transgenic models of Alzheimer's disease (AD) have made significant contributions to our understanding of AD pathogenesis, and are useful tools in the development of potential therapeutics. The fruit fly, *Drosophila melanogaster*, provides a genetically tractable, powerful system to study the biochemical, genetic, environmental, and behavioral aspects of complex human diseases, including AD. In an effort to model AD, we over-expressed human APP and BACE genes in the Drosophila central nervous system. Biochemical, neuroanatomical, and behavioral analyses indicate that these flies exhibit aspects of clinical AD neuropathology and symptomology. These include the generation of Aβ_40_ and Aβ_42_, the presence of amyloid aggregates, dramatic neuroanatomical changes, defects in motor reflex behavior, and defects in memory. In addition, these flies exhibit external morphological abnormalities. Treatment with a γ-secretase inhibitor suppressed these phenotypes. Further, all of these phenotypes are present within the first few days of adult fly life. Taken together these data demonstrate that this transgenic AD model can serve as a powerful tool for the identification of AD therapeutic interventions.

## Introduction

Alzheimer's disease (AD) is a progressive neurodegenerative disorder and is the most common cause of dementia in the developed world [1]. The pathological features of AD include the presence of amyloid plaques, neurofibrillary tangles, and loss of neurons, primarily in the cerebral cortex and hippocampus [2]. Amyloid plaques are extracellular deposits mainly composed of a small peptide (∼4 kD) called β-amyloid (Aβ), surrounded by dystrophic neurites, reactive microglia and astrocytes [3]. Several lines of evidence support the amyloid hypothesis of AD, according to which Aβ plays the central role in initiating the AD pathogenic cascade [4].

Aβ peptides are generated by proteolytic processing of the β-amyloid precursor protein (APP) through sequential proteolysis by β- and γ-secretases in the amyloidogenic processing pathways [5]. This pathway is initiated when APP undergoes proteolytic cleavage by β-secretase, encoded by the *BACE* gene. This cleavage produces a soluble extracellular/lumenal fragment of APP (sAPPβ) and a membrane spanning C-terminal fragment (βCTF/C99). The γ-secretase complex then cleaves βCTF to produce Aβ peptides and the APP intracellular domain (AICD) [5]. Aβ peptides of a variety of lengths are produced but Aβ_40_ and Aβ_42_ are the major isoforms produced in the central nervous system (CNS). Compared to Aβ_40_, Aβ_42_ is more prone to oligomerization and has been shown to be more neurotoxic [6].

APP also undergoes an alternative proteolytic processing pathway termed the non-amyloidogenic pathway. In this pathway, α-secretase initially cleaves APP, rather than β-secretase, to produce a soluble extracellular/lumenal fragment of APP (sAPPα) and a membrane spanning C-terminal fragment (αCTF/C83). Again, the γ-secretase complex then cleaves αCTF to produce the P3 peptide and AICD [5].

APP proteolysis is an important step towards development of AD. Therefore, it is important to identify genes and pharmaceuticals that modulate APP metabolism and Aβ production and clearance. Developing *in vivo* disease models has proven crucial to illuminating disease mechanisms, since *in vitro* studies do not always represent the natural physiological complexity of the tissue and/or organism. In particular, the fruit fly, *Drosophila melanogaster*, has been tremendously important and influential in furthering our understanding of the mechanisms of many forms of neurodegenerative diseases, including AD [7], [8], [9], [10], [11].

*Drosophila* endogenously express orthologues to the human *APP* [12], α-secretase [13], [14], and γ-secretase [15], [16], [17], [18]. Recently, a functional *Drosophila* homolog of the BACE (β-secretase) family of proteins has also been identified [19]. Though the *Drosophila* homolog to human *APP*, *Appl*, does not contain significant sequence similarity within the Aβ region of human APP [12], there is recent evidence suggesting that the fly Appl protein is also capable of generating neurotoxic Aβ-like fragments when the fly Appl and fly β-secretase proteins are overexpressed in *Drosophila* tissues [19]. These features position the fly as an attractive model to further study the evolutionarily conserved functions of these endogenous proteins.

Even though flies express orthologues of APP and secretase proteins, other *Drosophila* models of AD have been generated that express the human genes to gain insight into mechanism of disease and to illuminate potential therapeutic approaches. Many of these *Drosophila* AD models express the toxic human Aβ_42_ to study its effects on a molecular and behavioral level [9], [20], [21], [22], [23], [24]. These models have been useful in further dissecting the basic mechanisms behind human disease phenotypes such as amyloid deposits, learning and memory deficiences, and premature death. This method of expressing wild-type Aβ and disease associated Aβ sequence variants is useful for modulating the disease phenotype after disease progression has begun. Fewer reports have been published that rely on human APP proteolytic processing in the *Drosophila* CNS to generate Aβ oligomers [11], even though it has been shown that the endogenous fly secretases can process the human form of APP [7], [8].

The targeted expression of human AD genes in the fly has been used previously, with a focus on expression in the retina, wing, and the nervous system [7], [8], [11]. Here we express the human *APP* and *BACE* genes within the developing nervous system of *Drosophila*. This results in a model that displays very similar pathology to human Alzheimer's patients, including accumulation of Aβ-containing puncta in their brains, decreased dendritic and axonal fields in areas of the brain important for learning and memory, and memory deficits. A significant advantage of the model we describe is that these neuropathologies and memory defects are evident within days. We demonstrate that all of these phenotypes can be pharmacologically suppressed by the γ-secretase inhibitor L-685,458, illustrating the efficacy of this model for the rapid testing of small molecules for therapeutic intervention *in vivo*.

## Results

Expression of the human *APP* gene alone or in combination with the human β-secretase (*BACE*) gene exclusively in the developing fly nervous system was accomplished using the GAL4/UAS system [25]. Specifically, we utilized *elav-GAL4*, which drives protein expression throughout the fly CNS [26].

**Figure 1:**
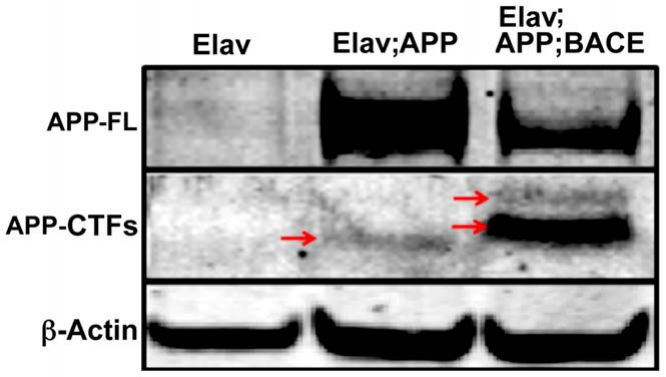
Transgene expression and proteolytic processing. Western blot analysis of human APP and fly β-actin detected in fly head lysates of: 1) *elav*; *+*; *+* heterozygous flies 2) *elav*; *APP*; + heterozygous flies, and 3) *elav*; *APP*; *BACE* heterozygous flies. APP-FL (full length APP, ∼110 kD) and APP-CTFs (C terminal fragments, ∼10–12 kD) were detected using A8717 (Sigma). Red arrows indicate α-CTF (lane 2) and β-CTFs (lane 3). A fly β-actin specific antibody was utilized (Abcam).

Using Western blot analysis, full-length APP is detected in the brains of flies expressing either APP or APP/BACE under control of the *elav* promoter, but APP is not detected in control flies lacking the APP transgene (Figure 1), as expected in the absence of the *elav-GAL4* driver. In brain tissue from *elav*; *APP*; *BACE* heterozygous flies, detection of BACE (Figure S1D) andβ CTFs are evidence of BACE expression and β-secretase activity (Figure 1, red arrows in third lane) respectively. β-secretase activity is not evident in *elav*; *APP* heterozygous flies, since only the α-secretase generated αCTFwas detected (Figure 1, red arrow in second lane).

To determine if the βCTFs were further processed by γ-secretase we measured Aβlevels by ELISA and by Western blot. AβELISA results demonstrate that *elav*; *APP*; *BACE* heterozygous flies produce signficantly higher levels of Aβ_40_ and Aβ_42_ than those not expressing human APP or BACE (Table 1). When *elav*; *APP*; *BACE* heterozygous flies are raised on food containing 100 nM L-685,458, a γ-secretase transition state inhibitor, Aβlevels are undetectable (Table 1). This indicates that γ-secretase activity is inhibited successfully in these flies, as is the subsequent production of Aβ. This result was confirmed by Western blot analysis of *elav*; *APP*; *BACE* fly heads, which demonstrate decreased Aβ levels in the L-685,458 treated flies compared to the DMSO (vehicle) raised *elav*; *APP*; *BACE* heterozygous flies (Figure S1C and S1F). The βCTF is the substrate for γ-secretase cleavage in APP amyloidogenic processing. Inhibition of γ-secretase activity should result in increased CTF levels. Consistent with this, we observed increased βCTF levels in the *elav*; *APP*; *BACE* heterozygous flies raised on L-685,458 containing food compared to those raised on DMSO (Figures S1B and S1F), as well as a modest increase in full length APP levels in flies raised on L-685,458 (Figures S1A and S1F). Treatment with either DMSO or L-685, 458 did not alter expression of BACE (Figures S1E and S1F). Therefore, CNS expression of human APP and BACE recapitulates APP amyloidogenic processing observed *in vitro* and in rodent transgenic AD models.

**Table 1:** Aβ levels.

Genotype	Treatment	Aβ_40_ (pg/mL)	Aβ_42_ (pg/mL)
*elav*; *+*; *+*	–	36±14	<5
*elav*; *PP*; *BACE*	DMSO	110±8	114±22
*elav*; *APP*; *BACE*	L-685,458	<5	<5

Aβ levels detected in fly head lysates (genotypes indicated) by ELISA.

**Figure 2:**
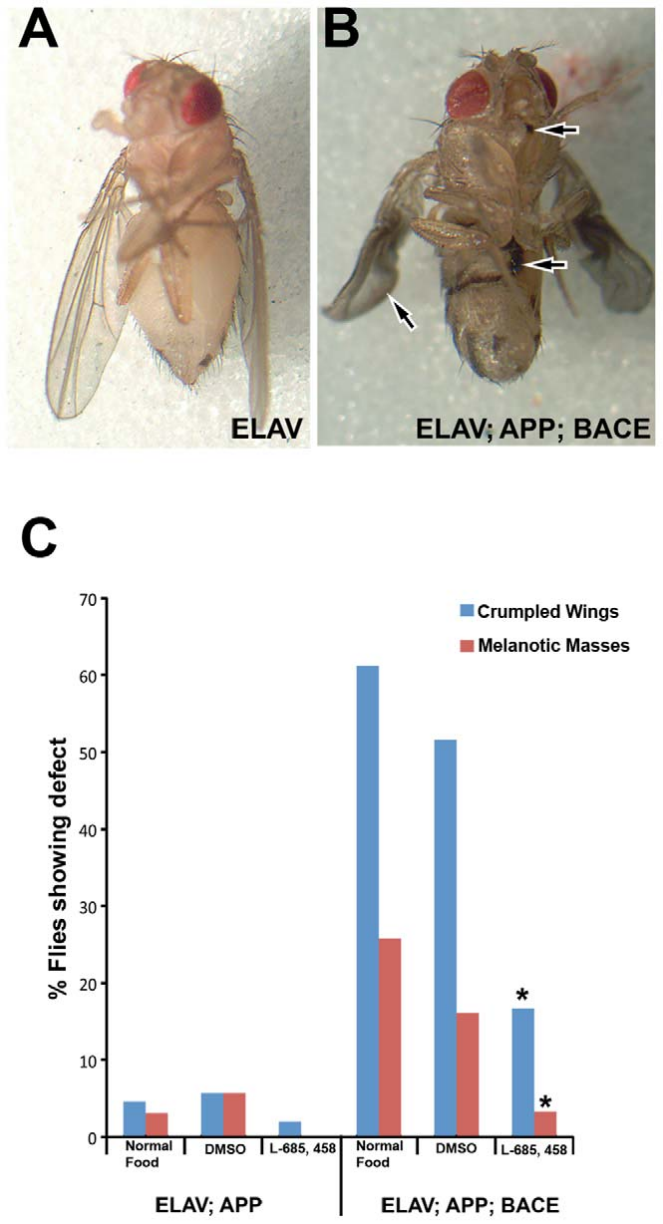
External morphology and longevity in AD flies. **A**) *elav*; *+*; *+* heterozygous fly one day after eclosion showing no defects on ventral proboscis, ventral abdomen, or in wings. **B**) Arrows indicate regions of the *elav*; *APP*; *BACE* heterozygous fly one day after eclosion showing melanotic masses on ventral proboscis, ventral abdomen, and curled wings. **C**) Quantification of the external defects shown in (B). n>50 in each case. Genotypes and treatments indicated. * indicates *p<.001* between DMSO and L-685,458.

In this model APP and BACE are expressed continuously during fly development. Upon adult eclosion, we observed two distinct morphological abnormalities in these flies: crumpled wings, and the presence of melanotic masses on both the abdomen and proboscis (arrows in Figure 2B). Crumpled wings were observed in 61% of all *elav*; *APP*; *BACE* heterozygous flies, while necrotic tissue was observed in 26% of the same genotype (Figure 2C). These phenotypes are observed in flies expressing human APP alone but at an approximately tenfold reduced penetrance compared to flies expressing both human APP and human BACE (Figure 2C) consistent with the idea that the phenotypes are dependent upon the expression of human BACE and amyloidogenic APP processing. More evidence to support this idea is that *elav*; *APP*; *BACE* heterozygous flies raised on food containing L-685,458 display significantly fewer crumpled wings (*p = 0.001*) or necrotic tissue (*p = 0.001*; Figure 2C). Specifically, L-685,458 treatment reduced the occurrence of crumpled wings to 17% from 52% observed in *elav*; *APP*; *BACE* flies treated with vehicle (DMSO). Additionally, L-685,458 treatment reduced the presence of melanotic masses to 3% from 16%.

We next compared the longevity of flies expressing human APP and human BACE to those flies only expressing human APP or human BACE alone. We created both survival and hazard plots for each genotype for analysis (Figure S2). We calculated the median survival time as the time when the survivor function equals 50%. As some flies were censored during the experiment (those that flew away or died of unnatural causes), median survivorship reflects a more reliable metric than the mean survival time.

The median survival time for *elav*; *APP*; *BACE* heterozygous flies was 32 days, compared to 42 days for *elav*; + heterozygous flies, and 56 days for *+*; *APP*; *BACE* heterozygous flies (Figure S2A). We found that until day 45, *elav*; *APP*; *BACE* heterozygous flies consistently had a lower probability of survival (Figure S2A). We found a statistical difference in survival between *elav*; *APP*; *BACE* heterozygous flies and controls (*p<0.0001*), suggesting that these flies displayed decreased survival, but this effect was limited to only young adults. While we found that there was no significant difference in the probability of survival between *elav*; *BACE*; *+* heterozygous flies and controls (*p = 0.1207*) (Figure S2D), we did find a significant difference in the probability of survival between *elav*; *APP*; *+* heterozygous flies and controls (*p<0.0001*) (Figure S2C). The median survival time of *elav*; *APP* heterozygous flies was 6 days, while the median survival time for the *+*; *APP* heterozygous and *elav*; *+* heterozygous flies was 38 and 45, respectively (Figure S2C). Again, the effect on survival was limited to only young adults. Finally, we compared the survival time for *elav*; *APP*; *BACE* heterozygous flies fed on DMSO and L-685, 458, and found no significant difference in the probability of survival (*p = 0.5038*). Taken together, these results suggest that while there is an effect on survival in the *elav*; *APP*; *BACE* heterozygous genotype in young adult flies, this effect does not require either BACE or γ-secretase function, and therefore most likely does not represent an effect of human Aβ accumulation in these flies.

**Figure 3:**
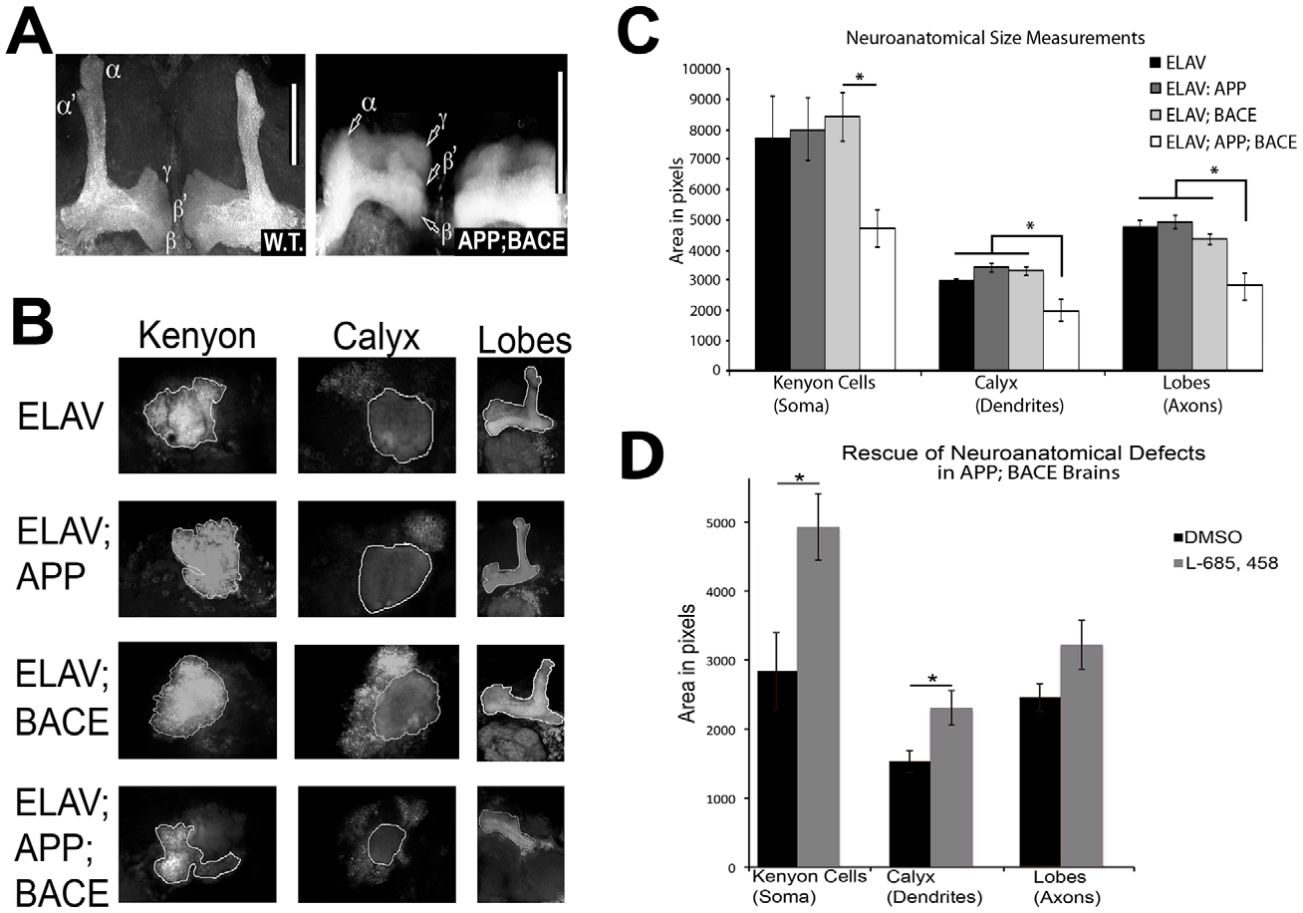
Defects in AD fly neuroanatomy. **A**) Mushroom body from *elav-CD8-GFP*; *+*; *+* heterozygous fly brain (left) displays normal axonal lobes. The same region in *elav-CD8-GFP*; *APP*; *BACE* heterozygous fly brain (right) displays significantly altered axonal lobes. These lobes are labeled. Note the complete loss of α′ lobe in *elav-CD8-GFP*; *APP*; *BACE* heterozygous fly brain. Scale bars indicate 50 µm in both panels. **B**) Examples of Kenyon cell (KC) substructures measured in the indicated genotypes. Kenyon indicates KC cell soma. Calyx indicates KC dendritic field. Lobes indicate KC axonal field. **C**) Neuroanatomical surface area measurements. Genotypes and substructures indicated. * indicates *p<0.05*, with exact values listed in text. **D**) Pharmacological rescue of neuroanatomical surface area decreases in *elav-CD8-GFP*; *APP*; *BACE* heterozygous fly brains. Treatments indicated. * indicates *p<0.05*, ANOVA analysis with exact values listed in text. Error bars represent standard error in each case.

We next compared the gross anatomical features of whole brains from *elav-CD8*; *+*; *+* heterozygous flies and *elav-CD8*; *APP*; *BACE* heterozygous flies (Figures S3A and S3B, respectively). We co-expressed a membrane tagged form of GFP (CD8-GFP) in the CNS of flies with the indicated transgenes to fluorescently visualize whole brain anatomy. *elav-CD8*; *APP*; *BACE* heterozygous flies displayed evidence of significant neuroanatomical changes compared to *elav-CD8*; *+*; *+* heterozygous flies and *elav-CD8*; *APP*; *+* heterozygous flies (Figures S3A and S3B). A number of specific brain structures/regions are altered including the the mushroom body (Figure 3A), the antennal lobes, and the optic lobes (Figure S3). The mushroom bodies are axonal bundles involved in learning and memory behavior in multiple experimental paradigms [27], [28], [29], [30], [31]. These axons extend from a population of neurons that consist of three distinct groups, which give rise to a final adult structure consisting of 5 projections to the α, α′, β, β′, and γ lobes (Figure 3A) [32]. These structures are significantly smaller in *elav-CD8*; *APP*; *BACE* heterozygous flies compared to controls (Figure 3A). In multiple cases axons from the mushroom body extending to the α and α′ lobes are either significantly shorter than controls, and/or missing completely (Figure 3A).

In order to quantify these observations we measured the sizes of the Kenyon cells (soma), calyx (dendritic field), and lobes (axonal bundles) of the mushroom body neurons (Figure 3B). *elav-CD8*; *APP*; *BACE* heterozygous flies display significant reductions in the sizes of the calyx (*p = 0.005*) and the lobes (*p = 0.005*) compared to *elav-CD8*; *APP*; + heterozygous flies, *elav-CD8*; *BACE*; *+* heterozygous flies, or *elav*; *+* heterozygous control flies (Figures 3B, C). However, *elav-CD8*; *APP*; *BACE* heterozygous flies do not display a significant (*p = 0.099*) decrease in Kenyon cell soma size compared to either *elav*; *+* heterozygous flies, or *elav*; *APP*; *+* heterozygous flies (Figure 3C). *elav-CD8*; *APP*; *BACE* heterozygous flies do show a significant difference in size compared to *elav*; *BACE*; *+* heterozygous flies (*p = 0.018*). However, Kenyon cell fields from *elav*; *BACE*; *+* heterozygous flies do not show a significant different between either *elav*; *APP*; *+* heterozygous flies or *Elav*; *+* heterozygous flies themselves (*p* = 0.393 and 0.989 respectively).

Culturing *elav-CD8*; *APP*; *BACE* heterozygous flies on food containing the common drug vehicle DMSO resulted in a decrease in Kenyon cell size as compared to the same genotype cultured on medium containing no DMSO, indicating that DMSO has a negative effect on these neurons in this genetic background. This is consistent with previous literature suggesting that DMSO has deleterious effects in flies as well as other systems [33], [34], [35]. However, when *elav-CD8*; *APP*; *BACE* heterozygous flies were raised on food containing L-685,458 dissolved in DMSO we observe a significant increase in the size of the Kenyon cell soma (*p = 0.03*) and calyx (*p = 0.03*) compared to *elav-CD8*; *APP*; *BACE* heterozygous flies raised on DMSO alone (Figure 3D). However, a significant increase in the axonal fields upon L-685,458 treatment was not observed (*p = 0.14*; Figure 3D). In conclusion, *elav-CD8*; *APP*; *BACE* heterozygous flies have reduced dendritic and axonal fields in structures involved in learning and memory. Further, these reductions require the expression of human APP and BACE, as well as the proper function of the γ-secretase complex, consistent with the idea that these neuroanatomical phenotypes are dependent upon amyloidogenic APP processing.

**Figure 4:**
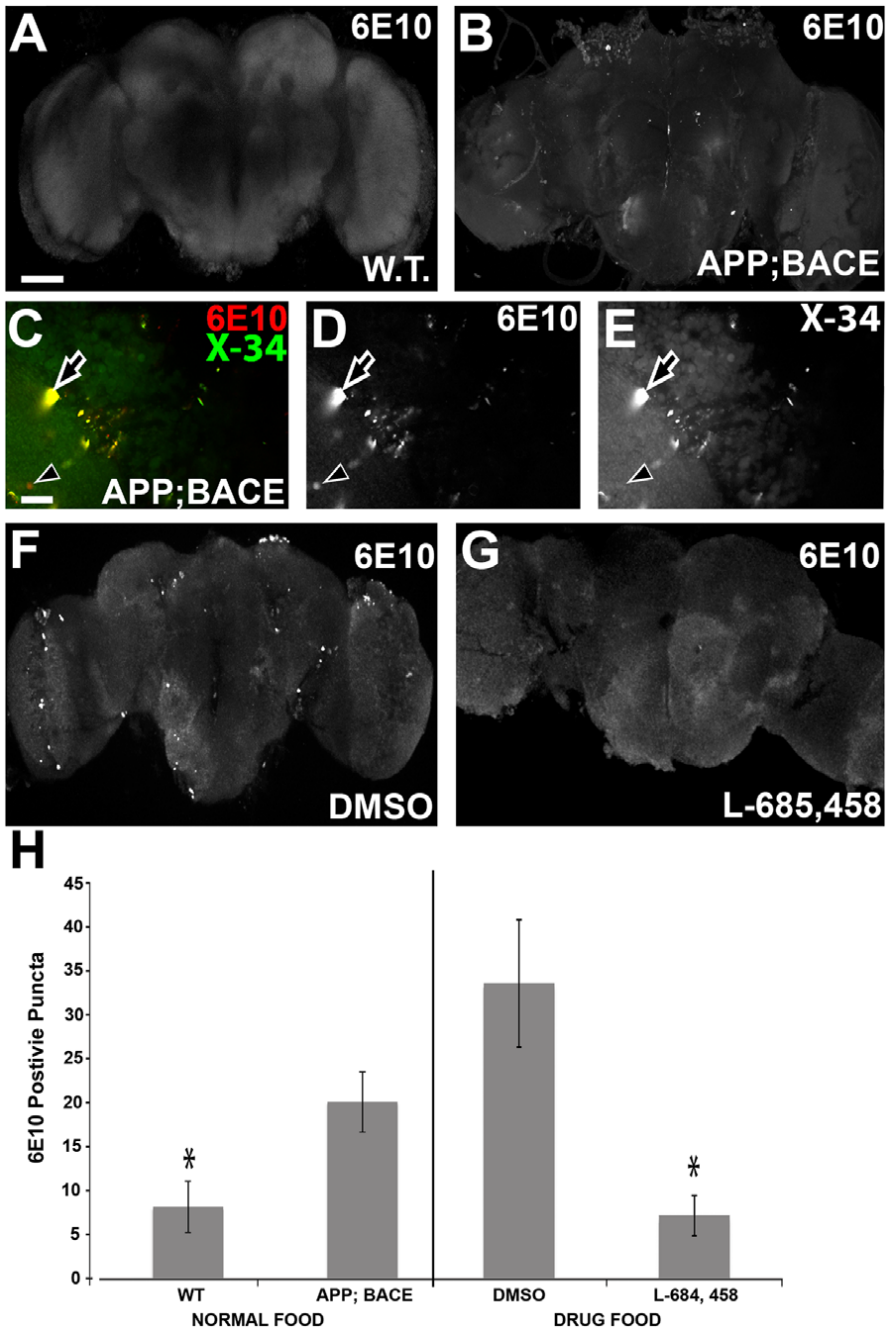
Aβ and amyloid detection in CNSs. **A**) *elav*; *+*; *+* heterozygous fly brain six days after eclosion shows minimal immunoreactivity to 6E10. Scale bar indicates 100 µm for panels A, B, F, and G. **B**) *elav*; *APP*; *BACE* heterozygous fly brain six days after eclosion shows significant 6E10 immunoreactive puncta. **C**) Co-localization of puncta to 6E10 and X-34 immunoreactivity in *elav*; *APP*; *BACE* heterozygous fly brains. High magnification (600×) of 6E10 puncta (red) from cortical area of fly brain show a majority of puncta co-localized with X-34 stain (green). Arrow indicates one example of 6E10 colocalizing with X-34 positive puncta. Arrowhead indicates one example of 6E10 that does not colocalize with X-34 puncta. Scale bar indicates 20 µm for C, D, and E. **D**) 6E10 positive puncta (white) from panel C. **E**) X-34 positive puncta from panel C. **F**) *elav*; *APP*; *BACE* heterozygous fly brain six days after eclosion from flies treated with DMSO (vehicle) shows significant 6E10 immunoreactive puncta. **G**) *elav*; *APP*; *BACE* heterozygous fly brain six days after eclosion from flies treated with L-685,458 (γ-secretase inhibitor) shows minimal 6E10 immunoreactive puncta. **H**) Quantification of panels A and B (left) and F and G (right). Error bars represent standard error. * indicates *p<0.05*. *n* = 3–5 fly brains.

Having observed significant neuroanatomical changes in the *elav-CD8*; *APP*; *BACE* heterozygous flies, we investigated whether these brains also displayed Aβpositive puncta. Labeling *elav-CD8*; *APP*; *BACE* heterozygous fly brains with the anti-Aβ antibody, 6E10, we detected significantly more 6E10 positive puncta in *elav-CD8*; *APP*; *BACE* heterozygous flies as compared to *elav-CD8*; *+*; *+* heterozygous controls (Figure 4B compared to 4A, Figure 4H). These 6E10 reactive puncta are found in the same brain regions as we observed significant neuroanatomical changes. Figure S3C shows a higher resolution image of 6E10 immunoreactive puncta in region near the Kenyon cell soma. Thioflavin S confirms the presence of amyloid puncta in the same cortical areas as the 6E10 antibody of *elav*; *APP*; *BACE* heterozygous flies (Figure S3D). To confirm that the 6E10 puncta we observe could co-localize with a stain for amyloid, we stained these brains with both 6E10 and X-34, a fluorescent Congo red derivative. X-34 has previously been shown to detect a variety of amyloid structures in brain tissue [36]. We analyzed multiple high magnification (600×) images of *elav-CD8*; *APP*; *BACE* heterozygous fly brains to determine the frequency of colocalization of 6E10 and X-34. We observe that on average 84.2% of X-34 puncta co-localize with 6E10, while 59.2% of 6E10 puncta co-localize with X-34 stain in *elav*; *APP*; *BACE* heterozygous fly brains (Figures 4C–E), suggesting that our 6E10 immunoreactive puncta contain material in a β-pleated sheet conformation.

Culturing *elav-CD8*; *APP*; *BACE* heterozygous flies on L-685,458 resulted in a significant decrease in the number of 6E10 immunoreactive puncta compared to DMSO treated flies of the same genotype (Figures 4F, 4G, and 4H). These results further confirm the presence of Aβ positive puncta in the brains of the *elav-CD8*; *APP*; *BACE* heterozygous flies, and show that presence of these puncta is dependent on γ-secretase activity, as expected.

**Figure 5:**
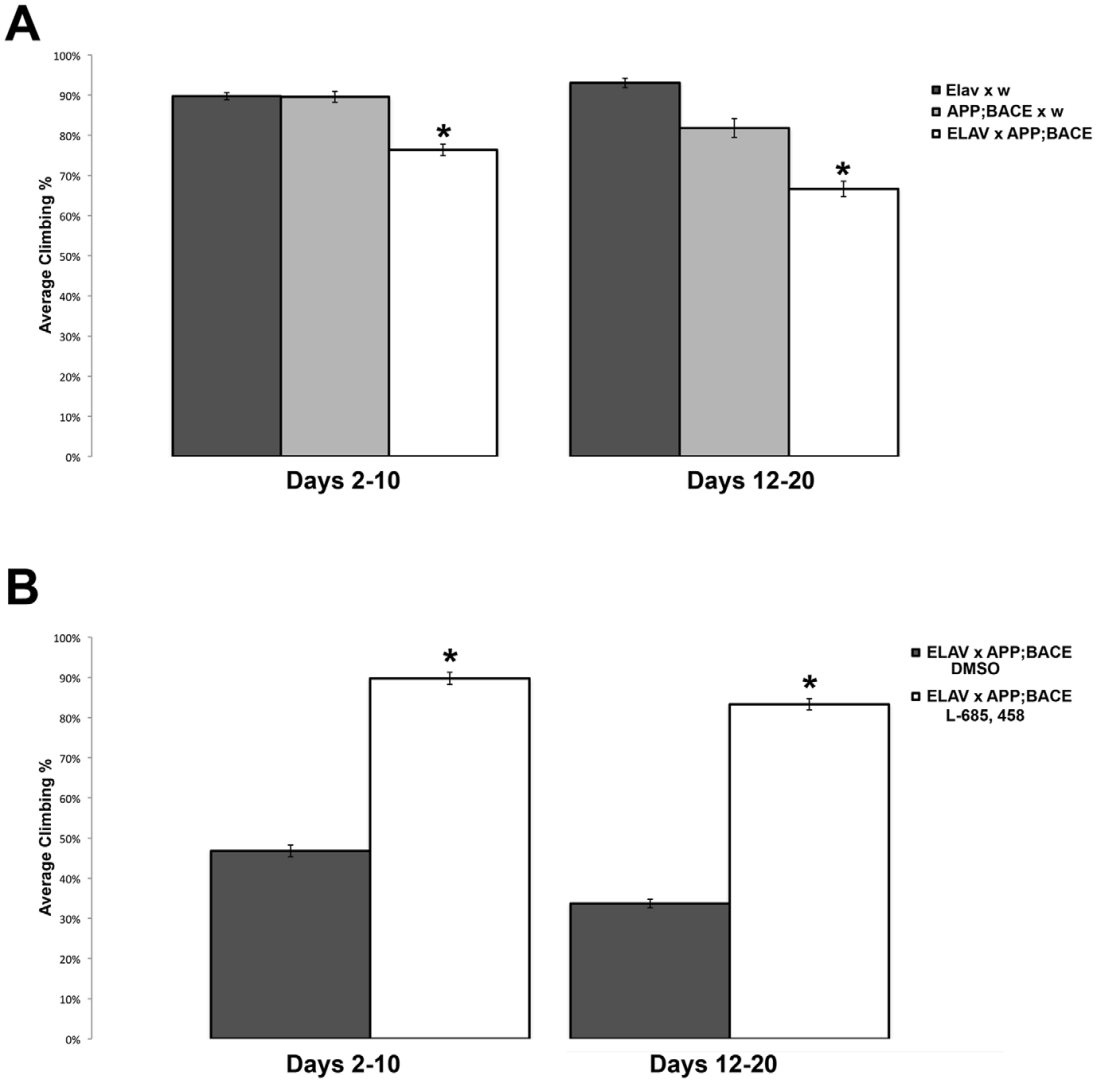
Motor reflex behavior. **A**) Climbing assay measurement of motor reflex behavior of *elav*; *APP*; *BACE* heterozygous flies, as compared to *elav*; *+*; *+* heterozygous flies and *w*; *APP:BACE* heterozygous flies. Parental strains indicated. **B**) Modulation of *elav*; *APP:BACE* heterozygous flies' motor reflex behavior by γ-secretase inhibitor, L-685,458. Genotypes and treatment indicated. * indicates *p<0.05*. *n* = 50–100 flies. Error bars represent standard error.

Having observed marked neuroanatomical and neuropathological changes in the *elav-CD8*; *APP*; *BACE* heterozygous flies, we wanted to determine if CNS function may be compromised. As an initial test of CNS function, we utilized a simple, yet powerful behavioral assay, the climbing assay [37]. This well-established assay has been previously used to assess nervous system dysfunction in fly models of multiple diseases, including AD [9]. Briefly, flies display a negative geotaxis response when given a mechanical stimulus. When tapped to the bottom of a vial, flies normally orient themselves rapidly and begin climbing to the top. By assaying the fly's ability to climb to the top of a vial in a set time period (18 seconds) we are able to compare broad nervous system function of reflex behavior between flies of different genotypes, or flies treated with different pharmacologic agents. When cultured on normal food, flies that express both human APP and human BACE (*elav*; *APP*; *BACE* heterozygous flies) show a significant decrease in their climbing ability compared to outcrossed control flies (Figure 5A). When cultured on food that contains L-685,458, the decrease in climbing ability is significantly rescued compared to flies cultured on food that contains vehicle (DMSO) control flies (Figure 5B). To rule out the individual effect of APP and BACE on this behavior, we repeated these experiments expressing only human APP (*elav*; *APP*; *+* heterozygous flies) or only human BACE (*elav*; *+*; *BACE* heterozygous flies) in the developing CNS. In both cases, we observed no significant difference in flies expressing human APP or human BACE alone compared to the appropriate transgenic outcrossed controls (Figures S4A and S4B).

Memory loss is the defining symptom of AD. To test for deficits in learning and memory in our AD model, we performed the conditioned courtship suppression assay [38]. This assay is ethologically based and capable of measuring both learning and memory in individual flies [39]. Courtship conditioning is a form of associative learning in *Drosophila*, where male courtship behavior is modified by exposure to a previously mated female that is unreceptive to courting [38], [40]. Thus, after a training period of 1 hour of courting a mated female, virgin males suppress their courtship behavior(s) even during subsequent exposure to receptive virgin females for 1–3 hours [38], [41], [42], [43].

**Figure 6:**
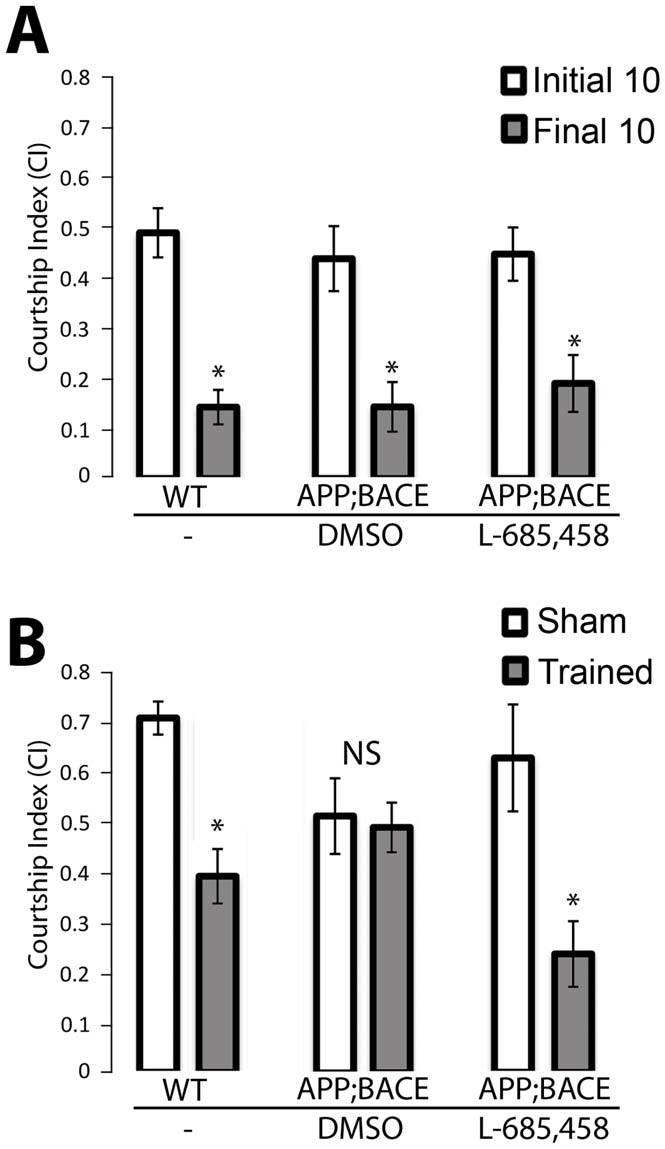
Learning and memory behavior. **A**) Panel denotes learning ability during the first 10 minutes (white columns) and last 10 minutes (grey columns) of the courtship suppression assay training phase. Treatments are indicated and WT indicates Canton S. Note normal learning response of *elav*; *APP*; *BACE* heterozygous flies raised on either DMSO or L-685, 458. **B**) Panel denotes immediate recall memory (0–2 minutes post-training) of trained flies (white columns) as compared to sham trained flies of matching genotypes and age (grey columns). *elav*; *APP*; *BACE* heterozygous flies treated with DMSO (vehicle) show no significant difference between trained and sham trained flies, indicating no immediate recall memory of training. This memory defect was rescued by treating flies with γ-secretase inhibitor, L-685,458. Error bars represent standard error. * indicates *p<0.05*. *n*≥18 for panels A and B.

To determine effects on learning in *elav*; *APP*; *BACE* heterozygous flies, male flies were placed in a courtship chamber with a previously mated (unreceptive) wild type (Canton S) female for 60 minutes. The amount of time the male spent performing courtship behavior was assessed during the first 10 minutes of this training and compared to the last 10 minutes of the training period. Wild type control flies showed a significant drop (*p = 0.0003*) in courtship behavior in the last 10 minutes of training as compared to the first 10 minutes of training (Figure 6A), indicative of an appropriate learning response. Flies that express human APP and human BACE (*elav*; *APP*; *BACE* heterozygous flies) also showed an appropriate learning response regardless of whether these flies were cultured on DMSO (*p = 0.0004*) or L-685,458 (*p = 0.0001*; Figure 6A). Importantly, this indicates that our *elav*; *APP*; *BACE* heterozygous flies are able to successfully perceive and interpret these sensory stimuli normally, and that they are able to alter their behavior appropriately (learn) in response to training.

There have been five phases of memory defined in *Drosophila*: immediate recall (0–2 minutes post-training), short term memory (up to 1 hour post-training), medium term memory (up to six hours), anesthesia-resistant memory (up to two days), and long term memory (up to 9 days) [44], [45]. We assayed the *elav*; *APP*; *BACE* heterozygous flies for immediate recall memory by transferring trained male flies to clean mating chambers with a receptive virgin female within 2 minutes of training. We then assayed their courtship behavior for 10 minutes. Trained wild type males showed a clear decrease in courtship activity as compared to parallel sham trained flies (*p = 0.00003*; Figure 6B), indicating a change in behavior consistent with normal immediate recall memory of training. However, *elav*; *APP*; *BACE* heterozygous flies cultured on DMSO showed no significant decrease (*p = 0.8*) in courtship behavior within 2 minutes of prior training compared to *elav*; *APP*; *BACE* heterozygous male flies unexposed to females (sham trained; Figure 6B). This indicates that though these flies are capable of learning, they are deficient in their immediate recall memory of this learning. Culturing *elav*; *APP*; *BACE* heterozygous flies on L-685,458 showed a clear decrease (*p = 0.005*) in courtship activity as compared to parallel sham trained *elav*; *APP*; *BACE* heterozygous flies cultured on this media (Figure 6B), indicating that L-685,458 can rescue the immediate recall memory defect normally associated with *elav*; *APP*; *BACE* heterozygous flies cultured on DMSO. This is interesting to note, particularly as culturing flies on L-685,458 does not fully rescue the decreased Kenyon neuron morphology in our *elav-CD8*; *APP*; *BACE* heterozygous flies.

## Discussion

*Drosophila* serves as a quick, efficient, and powerful *in vivo* tool for the analysis of multiple human diseases, including neurodegenerative diseases like AD. The main goal of developing such animal models is to use the abundant genetic, cellular, and behavioral tools available for these model organisms to discover and analyze novel molecules and genetic pathways that mediate the etiology, pathogenesis, and symptoms of human diseases. Because of the speed and sensitivity of *Drosophila*, these models can serve as excellent initial *in vivo* models to test for drug efficacy and possibly toxicity. Our results indicate that we have successfully created a Drosophila AD model that develops phenotypes rapidly and is sensitive to pharmacological rescue by a known inhibitor of γ-secretase. We show that these AD model flies can recapitulate amyloidogenic proteolytic processing of APP by β- and γ-secretase respectively, leading to the production of Aβ. We have also shown that presence of Aβ in the central nervous system of these flies can recapitulate some of the pathological, neuroanatomical and behavioral changes seen in AD patients. We suggest that this model will serve as a useful tool for future screening of genetic and pharmacologic modulators of APP proteolysis and Aβ production/toxicity/clearance.

Previously characterized *Drosophila* models of AD have largely relied on three basic approaches to investigate AD function (reviewed in [46]). The first set of approaches relies on analyzing the *Drosophila* homologs of the genes involved in AD. These include *Appl*, the *Drosophila* homolog of APP [19], [47], [48]; *tau*, the *Drosophila* homolog of *Tau* [49]; as well as *Drosophila* homologs of the γ-secretase complex genes *presenillin* [15], [17], *nicastrin* [50], *aph-1* [18], [51], and *pen-2* [18]. Expression of human *APP* rescues behavioral deficits associated with loss of fly *Appl*, showing that the human and fly proteins share a significant amount of functional homology [48]. By studying the basic functions of these fly genes, evolutionarily conserved functions of their mammalian counterparts can be determined, and this in turn can lead to a better understanding of the normal role of these genes and their gene products in neural development and neurodegeneration.

A second approach relies on the direct expression of human Aβ [9], [20], [21], [22], [23], [24], [52], [53], [54], [55], [56]. Examples include expression of Aβ in fly tissues to: identify genes associated with Aβ toxicity [52], validate computational predictions of mutant Aβ_42_ toxicity [24], test the pathological effect of different Aβ_42_ oligomerization states [22], test for pathological effects of Tau phosphorylation, and test for mitchondrial dysfunction [54], [55].

Finally, expression of the human forms of APP, BACE, Presenillin, and Tau [7], [8], [57], [58] has also been utilized, but to a lesser degree. Examples include the analysis of APP on axonal outgrowth after injury [47], testing the efficacy of pharmacological inhibitors [8] or peptide mimetics [11] on Aβ generation and toxicity, and testing the effect of Aβ aggregation on axonal transport defects [59].

Here, we expressed the human forms of APP and BACE exclusively within the developing nervous system of the fly allowing for the natural proteolytic processing of APP to occur in order to generate Aβ_42_. Expression was restricted to the CNS by using the *elav-GAL4* fly stock. This reagent places the yeast Gal4 protein under the control of the *elav* (*embryonic lethal, abnormal vision*) genomic enhancer region on the X chromosome [25]. *elav* encodes for an RNA binding protein that is expressed in all post-mitotic neurons [60], and has recently been shown to be expressed in embryonic glial cells, but not larval or adult glia [61]. Though other *Drosophila* models have similarly expressed both of these human proteins to model AD in the fly, these models have largely restricted their analysis to the developing retina [8] and wing tissues [7], [8]. In the retina, Greeve and colleagues observe that expression of human APP resulted in more neurodegeneration than the co-expression of human APP and human BACE [8]. They postulated that this suprising difference was due to the claveage of APP by a putative δ-secretase when BACE was not expressed. δ-secretase cleaves APP 12 residues N terminal of the β-secretase site. The long Aβ that results from δ- and γ-secretase cleavage produces more photoreceptor neurodegeneration. Interestingly, in our study the consequences of human APP and human BACE co-expression far outweigh those observed by expression of human APP alone in the CNS. We found no evidence of APP δ-secretase cleavage in our Western blot results (Figure 1 & Figure S1). This may suggest that δ-secretase expression is high in the retina and lower in the brain.

AD can be caused by increased APP expression levels. In humans, the APP gene is located on chromosome 21. Patients with Trisomy 21 (Down's Syndrome) invariably develop AD [62], [63]. Furthermore, APP locus duplications have also been identified in a small number of patients developing AD early in life [64]. This suggests that increased APP levels cause AD presumably by increased Aβ levels. Consistent with these clincial findings, we find that using a strong CNS promoter, *daughterless* (*da-GAL4*), to drive co-expression of human APP and human BACE results in pupal death (data not shown). Because of this effect, we utilized the *elav* promoter to drive transgene expression since it is weaker than the *da* promoter. Even with this relatively weak promoter, we observe strong biochemical, neuroanatomical, and behavioral effects. Using an even weaker promoter may result in more subtle phenotypes.

Recently, Sarantseva et al expressed both human APP and human BACE using the *elav-GAL4* driver at 29°C using standard yeast medium [11]. Consistent with the results presented here, these authors showed that these flies expressed APP, that this APP was processed successfully to generate Aβ monomers and oligomers, and that this Aβ accumulated in cortical regions of fly brains [11]. In their model, changes in neuroanatomy are also observed. Decreased mushroom bodies and antennal lobe sizes, consistent with the decreased mushroom body stuctures we observe in our model, are observed in 30 day old flies. These authors also report a defect in immediate recall learning (also called immediate recall memory) using an olfactory learning assay with 1–2 day old flies [11]. This defective immediate recall is consistent with our observations in 3 day old AD model flies, which display normal learning during the training period, but defective immediate recall memory. Because the olfactory learning task does not allow for testing of learning during training, these results suggest that in both of our models, immediate recall memory is defective in young adult flies.

Surprisingly, there are some significant differences between our two models. The neuroanatomical changes observed by Sarantseva et al are not apparent in young adult flies (two days old) but only in 30 day old flies [11]. This is in stark contrast to significant neuroanatomical changes we observe in six day old adult flies. The neuroanatomical changes we observe also occur concurrently with memory deficits, while this concurrence is not observed by Sarantseva et al. While both models observe strong neuropathological changes in the brains of *elav*; *APP*; *BACE* heterozygous flies, the flies described here also display phenotypes outside of the brain. Specifically we observe abnormal wing development and melanotic masses on the abdomen and proboscis, Sarantseva et al did not report such observations.

What could account for the differences we observe between our models? The calorie content of the fly food may be one cause. There are multiple recipes in use for fly media (http://flystocks.bio.indiana.edu/Fly_Work/media-recipes/media-recipes.htm). Each differ in the kind and amount of sugar(s) used. We used a standard medium containing molasses, which has a higher calorie content than the standard media containing sucrose or dextrose. Consistent with this, we have observed that reducing calorie intake in another Drosophila AD model reduces γ-secretase cleavage of APP (Chakraborty et al., in preparation).

Abnormal wing development was previously observed in flies expressing human APP in wings [7]. Melanotic masses have not been previously described when human APP or human APP and human BACE are expressed. These masses are an immune response in flies due to the localized buildup of hemocytes (invertebrate phagocytes) in the presence of tissue damage, necrotic tissue, infection, or altered self components [65]. Melanotic masses are formed due to the activation of the Toll pathway, which is the major effector of the innate immune response in flies [66]. The hemocyte response includes cell aggregation, phagocytosis, encapsulation of material (self or foreign), and the induction of the melanization cascade [65]. Though these melanotic masses appear in flies that only express human APP, the frequency of these melanotic masses increases almost tenfold when human BACE is co-expressed. Further, the frequency of these masses is significantly decreased when these flies are fed L-685,458, suggesting that these masses consist of Aβ or are induced by Aβ. This *Drosophila* immune response is reminscent of the inflammatory response that is invariably observed in human AD brain tissue [67], and may be in response to Aβ accumulation or Aβ-induced cellular/tissue damage. In mammals, Aβ has been shown to induce the inflammatory response via activation of Toll-like receptors 4 and 6 (TLR4/6) [68]. In light of a recent paper that suggests Aβ is an antimicrobial peptide in the innate immune response in humans [69], an alternative explanation to the role of Aβ in melanotic masses is that Aβ is an active participant in the innate immune response and thereby does not directly “cause” the response. Regardless of the mechanism of formation of these masses, they are apparent when flies first eclose from their pupal case, and may act as a proxy for cerebral Aβ accumulation.

In humans, there is a poor correlation between plaque load and cognitive function [70], [71], [72]. Therefore in addition to monitoring a proxy for cerebral Aβ accumulation, it is important to have a quick measure of CNS function. Our AD flies display a rapid decline in their reflex climbing behavior within the first 10 days after eclosion (Figure 5). As expected for any Aβ dependent process, this climbing defect can be rescued by treating the flies with L-685,458.

In each case, we see that there are significant effects of genetic background on climbing ability for each experimental genotype (Figure 5, and Figure S4). However, the only valid comparisons that can be made for these experimental genotypes must be made between experimental and outcrossed controls of the same groups. Thus, while *w*; *APP*; *+* heterozygous flies and *w*; *+*; *BACE* heterozygous flies show similar levels of climbing compared to *elav*; *APP*; *BACE* heterozygous flies, the fact that the *elav*; *APP*; *BACE* heterozygous flies contain both *UAS:APP* and *UAS:BACE* transgenes precludes comparison between these genotypes. The appropriate comparison must be made between *elav*; *APP*; *BACE* heterozygous flies and *w*; *APP*; *BACE* heterozygous flies, as it is the *w*; *APP*; *BACE* heterozygous genetic background that is included within our *elav*; *APP*; *BACE* heterozygous flies, and not the *APP*; *+* heterozygous genetic background alone. Thus, based on these genetic experiments, we can only conclude that induction of APP expression via the GAL4/UAS system is not detrimental to the climbing ability compared to either the *elav-GAL4* or *UAS:APP* backgrounds alone (Figure S4). This is also similar for BACE expression alone (Figure S4). However, when we combine the genetic background of *elav-Gal4* with the *UAS:APP*; *UAS:BACE* genetic background, we observe a significant decrease in climbing ability in the subsequent *elav*; *APP*; *BACE* heterozygous genetic background compared to either the *elav*; *+* heterozygous genetic background, or the *w*; *APP*; *BACE* heterozygous genetic backgrounds alone (Figure 5).

Finally, treatment of our AD model flies (*elav*; *APP*; *BACE* heterozygous flies) with the drug vehicle DMSO has deleterious effects on these flies, decreasing Kenyon Cell size, decreasing climbing reflex behavior, and increasing the number of Aβ puncta in fly brains (Figures 3, 4, 5). Previous literature has shown that DMSO can induce cytotoxicity in transgenic flies lines expressing *hsp70-lacZ* at 0.3% of dietary concentration [34]. Though the concentration of DMSO we used was lower at 0.1%, it is easy to imagine a scenario where a lower concentration of DMSO could have a deleterious effect on these flies is the cell's stress response is already activated or compromised, as is the case for our AD model flies (*elav*; *APP*; *BACE* heterozygous flies). Further, while the previous study examined hatchability, emergence, fecundity, reproductive performance, and hsp70 expression [34], our study focuses on neural function, which may be a more sensitive assay, especially within our model. In mice, DMSO has been shown to cause apoptosis throughout the central nervous system [33]. Further, cells from AD patients show increased endoplasmic reticulum calcium stores, a well-defined target of oxidative stress present in AD. DMSO treatment of these cells exaggerates H_2_O_2_ enhancement of this increased calcium storage [35], [73]. In each case analyzed, treatment with L-685, 458 in the presence of DMSO suppresses the phenotypes associated with our model, suggesting that though DMSO enhances the pathology in our fly model, suppression of the gamma secretase complex rescues this effect, in many cases back to relatively normal levels (ex. Figures 4 & 5). These data are consistent with a requirement for gamma secretase activity to induce these phenotypes.

In summary, the expression of human APP and human BACE genes in the Drosophila CNS results in biochemical, neuroanatomical, neuropathological, and behavioral changes that are reminiscent of clinical AD. We observe these changes early in the life of adult flies, and importantly, these changes are prevented with γ-secretase inhibitor treatment. Taken together, these measures provide a powerful and quick method to assess AD progression in our fly model, and may be used for the rapid testing of small molecules for therapeutic intervention.

## Materials and Methods

### Western Blot Analysis

For Western blot analysis, 15–20 fly heads were collected from respective genotypes and immediately lysed in RIPA buffer (50 mM Tris, 150 mM NaCl, 1% SDS, 1% NP-40, 0.5% deoxycholate, pH 8.0) containing a cocktail of protease inhibitors [Antipain(100 mM), Aprotinin (2 mg/ml), Benzamide (15 mg/ml), Chymostatin (100 mM), Leupeptin (100 mM), Pepstatin A (1 mM), PMSF (1 mM), Sodium Metabisulfite(0.1 nM)]. These lysates were stored at −80°C. As a control for BACE and APP expression, cell lysates of HEK293 cells were also prepared. The protein concentration of these fly head lysates was determined using the BCA Protein Assay Kit (Pierce, Inc.). According to the protein concentrations, samples for Western Blot were prepared using the 4× NuPage LDS sample buffer (Invitrogen, Inc.) containing 0.2% BME (β-Mercaptoethanol, Sigma Aldrich). Equal amounts of protein were loaded on to each well of NuPAGE 4–12% Bis Tris Gel. From the gel the proteins were transferred on to 0.25 µm PVDF (Immobilon FL) membrane (Millipore) using a semi-dry transfer apparatus. Blots were probed with the indicated antibodies and the target protein densitometry was normalized to β-actin densitometry using Odyssey Infrared Imaging system (LI-COR Biosciences).

### Antibodies

APP C-terminal antibody (A8717; Sigma Aldrich, Inc), BACE (ab2077, Abcam) monoclonal anti β-Actin (A5441, Sigma Aldrich, Inc), APP 6E10 (ab10146, Covance), goat anti-Rabbit IR-Dye800 CW (926–3211; LiCor) and/or goat anti-Mouse IR Dye 680 (926–3200; LiCor) were used as secondary antibodies.

### Immunohistochemistry

Adult and larval brains were dissected, fixed and prepared as described [74]. Adult and larval brains were dissected directly in fix. Brains were mounted in vectashield (Vector Labs, H-1000). All fluorescent imaging was done using an Olympus FluoView FV1000 laser scanning confocal microscope. Secondary antibodies for immunohistochemistry used were goat anti-mouse TRITC (# 115-116-072, 1∶150), goat anti-rabbit TRITC (# 111-116-144, 1∶250), goat anti-rabbit Cy5 (#111-176-144, 1∶1000), goat anti-mouse Cy5 (# 115-176-072, 1∶500). All secondary antibodies were from Jackson ImmunoResearch. Thioflavin S staining was performed as described [22]. X-34 staining was performed as described [36].

### Brain structure/puncta measurements and analysis

To measure the size of soma, calyx, and lobes, a membrane tagged form of GFP (CD8-GFP) was expressed in the nervous system under UAS control. Serial confocal microscope sections were obtained at 200× magnification, and the appropriate brain regions (Kenyon Cells, Calyx, Lobes) were stacked and pixels measured using Image J (http://rsbweb.nih.gov/ij/). Pixel measurements were generated using Image J. Five brains were analyzed for each genotype.

To count 6E10 positive puncta, 5 brains of each genotype were fully optically sectioned by confocal microscopy. An observer blinded to genotype scanned through each brain section and counted the number of 6E10 immunoreactive puncta in each section. These numbers were then averaged out for each genotype, and significance was determined by using an unpaired Student's t-test.

To count 6E10 and X-34 colocalization, 4 brains of from *elav-CD8*; *APP*; *BACE* heterozygous flies were imaged at 600× in areas near the mushroom body soma (as determined by GFP fluorescence). The number of 6E10 puncta were counted and averaged between each brain, as were the number of X-34 puncta. The number of puncta that were positive for both 6E10 and X-24, was determined by dividing the total number of co-labeled puncta by either 6E10 or X-34 positive puncta to derive % colocalization.

### Pharmacologic reagents used

γ-secretase transition state inhibitor, L-685,458, was purchased from Sigma Aldrich. 100 nM L-685,458 was used for preparing food vials for AD model flies. Drug or DMSO was added to water and mixed to homogeneity prior to preparing food. DMSO concentration was 0.1% in all cases. Flies were raised on food containing either drug or DMSO alone for their entire development and adult life (embryogeneis to death). After hatching, flies were maintained on DMSO or drug food containing L685, 458 dissolved in DMSO throughout their entire lifespan. No external yeast was added to this food at any point during the analysis.

### ELISA Analysis

Aβ_40_ and Aβ_42_ levels were determined using commercially available human Aβ specific ELISA kits (BetaMark, Covance, Dedham, MA) according to the manufacturer's instructions. BetaMark ELISA kits are resistant to interference from detergent and are compatible with tissue extracts containing low levels of SDS (<0.1%). RIPA buffer homogenates were diluted to a final SDS concentration of less than 0.1%. Aβ standards were assayed at in homogenization vehicle buffer at the same dilution as test samples. Lysates from an equal number of fly heads were compared across treatment or genotype. Lysates from 50 heads were used for each ELISA well. Two dilutions of each extract were prepared for assays. A 3∶5 dilution sample consisted of: 86 µL sample+124 µL PBS (0.6 dilution containing 0.06% SDS) further diluted 1∶1 in kit assay buffer (final 0.3 sample dilution containing 0.03% SDS in the well). A 3∶25 dilution sample consisted of: 37.2 µL sample+272.8 µL PBS (0.12 dilution containing 0.012% SDS) further diluted 1∶1 in kit assay buffer (final 0.06 dilution containing 0.006% SDS in the well). Blanks contained BSA protein in buffer. Fly head were dissected, immediately homogenized in RIPA buffer (with protease inhibitors) and then stored at −80°C.

### Drosophila Stocks and Genetics

All crosses and stocks were maintained at 25°C. Normal food consisted of a standard cornmeal, yeast, molasses recipe as follows: 120 g cornmeal (LabScientific FLY-8009-10), 48 g yeast (LabScientific 8030-5), 9 g agar, 120 ml molasses (LabScientific FLY-8008-4), 24 ml Tegosept (10% w/v methyl p-hydroxybenzoate in 95% ethanol), and 9.5 ml Propionic Acid) with 840 ml of water. Drug food was prepared adding the indicated drug to 17 ml of water and mixing thoroughly. Cornmeal, yeast, agar, molasses, tegosept, and propionic acid were then added to a final volume of 30 ml, and food was prepared as normal. Flies were cultured on drug food for their entire lifespan from embryogenesis to death. Drug food was changed every 3–4 days to ensure fresh exposure to drug.

The GAL4/UAS system was used for the overexpression of UAS transgenes in *Drosophila* as described [25]. BL# refers to Bloomington Stock Center stock number. Bloomington stocks *P{GawB}elav^C155^*, *P{UAS-mCD8::GFP.L}LL4*, *P{hsFLP}1*, w^*^ (BL#5146) and *P{GawB}elav^C155^* (BL#458) were used to drive transgene expression and are abbreviated in the text as elav and elav-CD8, respectively. The *P{UAS:APP}* and the *P{UAS:APP}*; *P{UAS:BACE}* [8] stock, referred to in the text as APP and APP; BACE respectively, were generous gifts from Rita Reifegerste. *w^1118^*; *P{UAS-BACE1.L}2* (BL#29877) referred to in the text as BACEused to examine the effects of the individual transgene. Bloomington stock *w^1118^* (BL#3605) was used to generate outcrossed controls and is referred to as *w* in the text. All transgenes are examined in the heterozygous state.

Wild type flies used for controls and training during the learning and memory assays were Canton S. All other controls are the appropriate transgenic controls, either lacking the GAL4 driver or UAS-linked transgene, as indicated.

### Behavioral testing and training

For all behavioral tests, flies were maintained at 25°C in a 12∶12 light∶dark cycle at 60% humidity. For longevity studies, flies were collected between 0–8 hours after eclosion, and were maintained in vials of 10 or fewer flies for their lifespan. These vials were kept on their side to minimize flies falling into the food at the bottom of the vial and perishing due to becoming stuck in the food. Any flies that died due to these food deaths or that flew away during the study were marked as censored from the longevity assay. Flies were checked every day during the relative light cycle and total number of living flies were recorded in each vial each day. A total of 50–140 flies of each genotype were assayed. Each vial maintained and tested had 10 or fewer flies. Due to certain flies flying away during the course of the study period and some flies dying in their food, every fly does not experience the event of interest; therefore, these flies were deemed censored observations and their observed data was classified as non-informative censoring. All statistical analyses for longevity were carried out using the statistical software package SAS 9.2. The Wilcoxon test of homogeneity (a test statistic that is often used in survival analysis to compare survival functions, especially when survivor functions tend to cross each other throughout points during the follow-up time) was used to determine statistical significance at the alpha level of 0.05 in all cases.

For climbing assays, a modified version of Le Bourg and Lints was used [37]. Flies were collected between 0–8 hours after eclosion and assayed every two days. Groups of 10 or fewer flies were maintained in vials kept on their side as above. During the climbing assay, these flies were transferred to a clean, empty vial and given 18 seconds to climb 5 cm. The number of flies that successfully reach the 5 cm line were recorded. Between 50–140 flies of each genotype were assayed for each vial of 10 or fewer flies. The average climbing success for days 2–10 and 12–20 for each genotype was binned, and significance was determined between genotypes by a one-way ANOVA analysis with genotype as the independent variable.

For courtship behavioral training, virgin male flies of the appropriate genotype were collected between 0 and 6 hours after eclosion and transferred to individual food vials. All flies were maintained at 25°C in a 12∶12 light∶dark cycle at 60% humidity. All behavioral tests were performed in a separate room maintained at 25°C and 60% humidity and illuminated under a constant 130 V white light Kodak Adjustable Safelight Lamp mounted above the courtship chambers. All behavior was digitally recorded using a Sony DCR-SR47 Handycam with Carl Zeiss optics. Subsequent digital video analysis of time spent performing courtship behavior was quantified using iMovies software (Apple). The total time that a male performed courtship activity was measured and scored. The Courtship Index (CI) was calculated as the total time observed performing courting behavior divided by the total time assayed, as described [38].

Virgin female wild type (Canton S) flies were collected and kept in normal food vials in groups of 10. Male flies were aged for 3 days before behavioral training and testing. All tests were performed during the relative light phase. Mated Cantons S females used for training were 5 days old, and observed to have mated with a Canton S male the evening prior to training. Virgin female Canton S targets used were 4 days old. Male flies were assigned to random groups the day of training, and assays were set up and scored blind. Male flies were transferred without anesthesia to one half of a partitioned mating chambers from Aktogen (http://www.aktogen.com) that contained a previously mated Canton S female in the other partitioned half. Males were allowed to acclimate for 1 minute, then the partition between the male and female was removed. Male flies were then trained for 60 minutes. After 60 minutes, male flies were transferred within 2 minutes without anesthesia to one half of a clean partitioned mating chamber that contained a virgin Canton S female in the other partitioned half. The partition was removed and the flies were recorded for 10 minutes. A total of 18–22 flies were scored for each genotype, both trained and sham. To determine significance among the same individuals for the learning phase of this assay, a two-tailed paired Student's t-test was performed. To determine significance among different individuals of the same gentotype a two-tailed unpaired Student's t-test was performed.

### Statistical Analysis

All statistical analysis was performed on PASWStatistics version 18.0 with the exception of the survival data described above. To determine significance between multiple different genotypes, a one-way ANOVA analysis was performed with Tukey posthoc analysis. Genotype is the independent variable. To determine significance between different measures of the same genotype, a two-tailed paired Student's t-test was performed. An unpaired Student's t-test was performed between 2 groups of different genotypes. To determine significance in phenotypic frequencies between different genotypes, a G test of goodness-of-fit was performed. Significance was determined at the 95% confidence interval.

## Supporting Information

Figure S1 **Western blot analysis of** ***elav*** **;** ***APP*** **;** ***BACE*** **heterozygous fly heads.** Fly treatments are indicated in figure for each lane. **A**) Detection of human APP in *elav*; *APP*; *BACE* heterozygous fly heads by A8717 anti-APP antibody (Sigma). Lane 1 shows cellular lysates from HEK293 cells stably expressing APP as a positive control. **B**) Detection of human CTFs in *elav*; *APP*; *BACE* heterozygous fly heads by A8717 anti-APP antibody (Sigma). Note that γ-secretase inhibitor, L-685,458 increases CTF levels. Lane 1 shows cellular lysates from HEK293 cells stably expressing APP as a positive control. **C**) Detection of human Aβ by 6E10 (Covance) *elav*; *APP*; *BACE* heterozygous fly head lysates. **D**) Detection of BACE (Abcam) in *elav*; *APP*; *BACE* heterozygote fly head lysates. Lane 1 shows cellular lysates from HEK293 cells stably expressing APP-Sw as a positive control. Note no BACE immunoreactivity was observed in *elav/w*; *+*; *+* fly head lysates, while BACE immunoreactivity was observed in *elav*; *APP*; *BACE* heterozygousfly head lysates. **E**) Detection of human BACE in *elav*; *APP*; *BACE* heterozygous fly heads. Lane 1 shows cellular lysates from HEK293 cells stably expressing BACE as a positive control. **F**) Quantification of panels A, B, C, and E Western blot signal intensity.
(TIF)

Figure S2 **Longevity and mortality analysis.** In each panel, longevity analysis is top chart and mortality chart is bottom panel. **A**) Longevity and mortality analysis of flies expressing human APP and human BACE (*elav*; *APP*; *BACE* heterozygous flies) compared to genetic background controls that either lack the driver (*w*; *APP*; *BACE* heterozygous flies) or UAS transgene (*elav*; *+*; *+* heterozygous flies). **B**) Longevity and mortality analysis of flies expressing human APP and human BACE (*elav*; *APP*; *BACE* heterozygous flies) raised on food containing DMSO (vehicle) or L-685, 458. **C**) Longevity and mortality analysis of flies expressing human APP alone (*elav*; *APP* heterozygous flies) compared to genetic background controls that either lack the driver (*w*; *APP*: *+* heterozygous flies) or UAS transgene (*elav*; *+*; *+* heterozygous flies). **D**) Longevity and mortality analysis of flies expressing human BACE alone (*elav*; *BACE*; *+* heterozygous flies) compared to genetic background controls that either lack the driver (*w*; *BACE*; *+* heterozygous flies) or UAS transgene (*elav*; *+*; *+* heterozygous flies).
(TIF)

Figure S3 **Whole brain neuroanatomy and Thioflavin S stain.** **A**) Membrane bound GFP fluorescence illuminates whole brain morphology in *elav-CD8-GFP*; *+*; *+* heterozygous fly brain from fly six days after eclosion. **B**) Dramatic changes in *elav-CD8-GFP*; *APP*; *BACE* heterozygous brain morphology six days after eclosion. **C**) High magnification (600×) of Thioflavin S positive puncta in cortical region of *elav*; *APP*; *BACE* heterozygous fly brain. Arrows indicate Thioflavin S positive puncta.
(TIF)

Figure S4 **Motor reflex behavior.** **A**) Expression of human APP alone does not change motor reflex behavior, as measured by the climbing assay. Parental strains listed. Error bars show standard error. No significant difference exists between *elav*; *APP*; *+* heterozygous flies and *elav*; *+*; *+* heterozygous flies in days 2–10 (ANOVA, *p = .601*) or in days 12–20 (*p = .677*). **B**) Expression of human BACE alone does not change motor reflex behavior (*elav*; *BACE*; *+* heterozygous flies). No significant difference was found between *elav*; *BACE*; + heterozygous flies and *w*; *BACE*; *+* heterozygous flies in days 2–10 (ANOVA, *p = .106*) or in days 12–20 (*p = .066*). Error bars represent standard error.
(TIF)
