# Supplementary figures and images for: Characterization of a Drosophila Alzheimer's Disease Model: Pharmacological Rescue of Cognitive Defects
Source: PLoS One. 2011 Jun 6;6(6):e20799. doi: 10.1371/journal.pone.0020799 (PMC3108982; doi:10.1371/journal.pone.0020799)

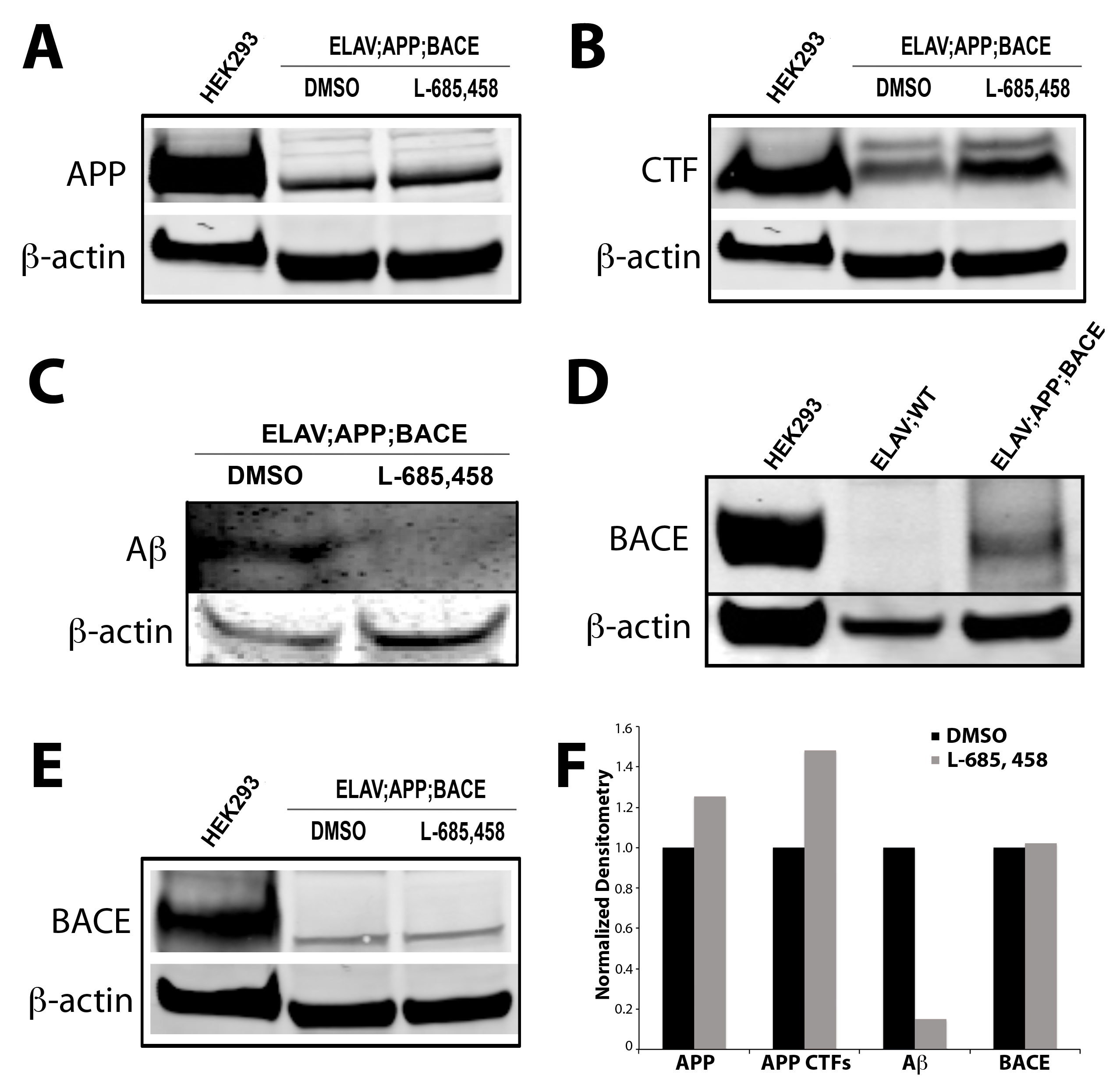

Supplement: Figure S1 — Western blot analysis of elav ; APP ; BACE heterozygous fly heads. Fly treatments are indicated in figure for each lane. A) Detection of human APP in elav; APP; BACE heterozygous fly heads by A8717 anti-APP antibody (Sigma). Lane 1 shows cellular lysates from HEK293 cells stably expressing APP as a positive control. B) Detection of human CTFs in elav; APP; BACE heterozygous fly heads by A8717 anti-APP antibody (Sigma). Note that γ-secretase inhibitor, L-685,458 increases CTF levels. Lane 1 shows cellular lysates from HEK293 cells stably expressing APP as a positive control. C) Detection of human Aβ by 6E10 (Covance) elav; APP; BACE heterozygous fly head lysates. D) Detection of BACE (Abcam) in elav; APP; BACE heterozygote fly head lysates. Lane 1 shows cellular lysates from HEK293 cells stably expressing APP-Sw as a positive control. Note no BACE immunoreactivity was observed in elav/w; +; + fly head lysates, while BACE immunoreactivity was observed in elav; APP; BACE heterozygousfly head lysates. E) Detection of human BACE in elav; APP; BACE heterozygous fly heads. Lane 1 shows cellular lysates from HEK293 cells stably expressing BACE as a positive control. F) Quantification of panels A, B, C, and E Western blot signal intensity. (TIF) [file pone.0020799.s001.tif]

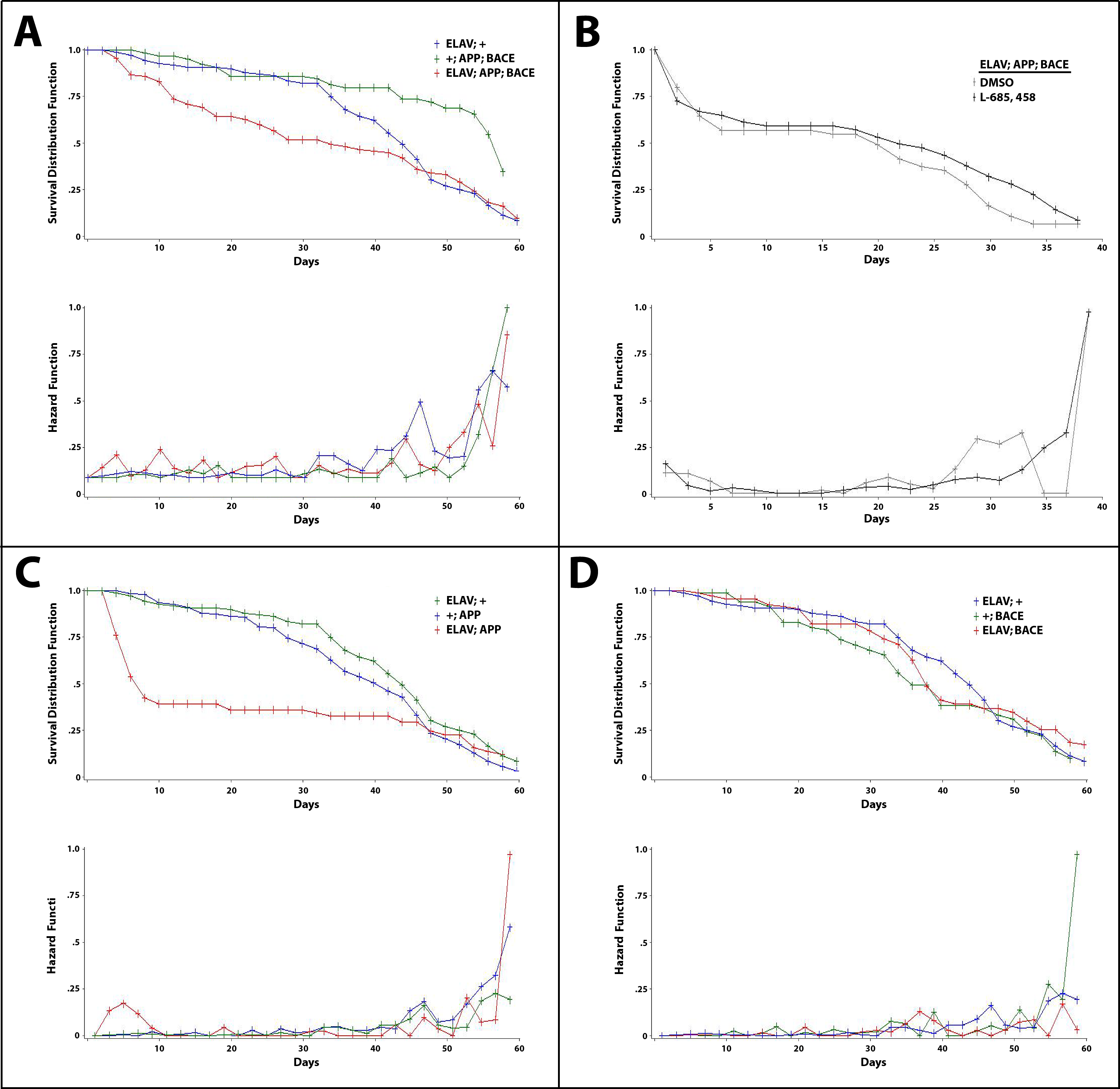

Supplement: Figure S2 — Longevity and mortality analysis. In each panel, longevity analysis is top chart and mortality chart is bottom panel. A) Longevity and mortality analysis of flies expressing human APP and human BACE (elav; APP; BACE heterozygous flies) compared to genetic background controls that either lack the driver (w; APP; BACE heterozygous flies) or UAS transgene (elav; +; + heterozygous flies). B) Longevity and mortality analysis of flies expressing human APP and human BACE (elav; APP; BACE heterozygous flies) raised on food containing DMSO (vehicle) or L-685, 458. C) Longevity and mortality analysis of flies expressing human APP alone (elav; APP heterozygous flies) compared to genetic background controls that either lack the driver (w; APP: + heterozygous flies) or UAS transgene (elav; +; + heterozygous flies). D) Longevity and mortality analysis of flies expressing human BACE alone (elav; BACE; + heterozygous flies) compared to genetic background controls that either lack the driver (w; BACE; + heterozygous flies) or UAS transgene (elav; +; + heterozygous flies). (TIF) [file pone.0020799.s002.tif]

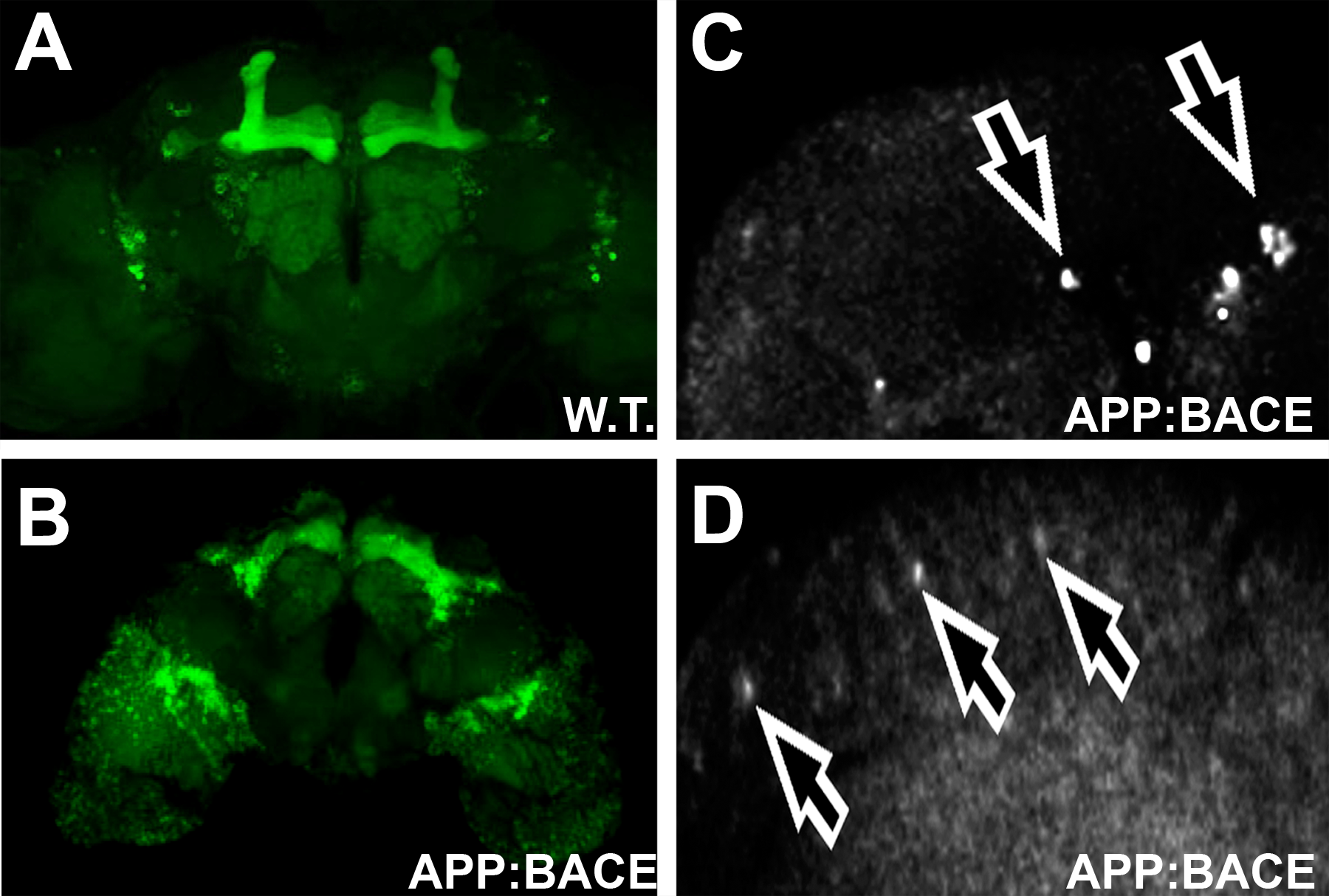

Supplement: Figure S3 — Whole brain neuroanatomy and Thioflavin S stain. A) Membrane bound GFP fluorescence illuminates whole brain morphology in elav-CD8-GFP; +; + heterozygous fly brain from fly six days after eclosion. B) Dramatic changes in elav-CD8-GFP; APP; BACE heterozygous brain morphology six days after eclosion. C) High magnification (600×) of Thioflavin S positive puncta in cortical region of elav; APP; BACE heterozygous fly brain. Arrows indicate Thioflavin S positive puncta. (TIF) [file pone.0020799.s003.tif]

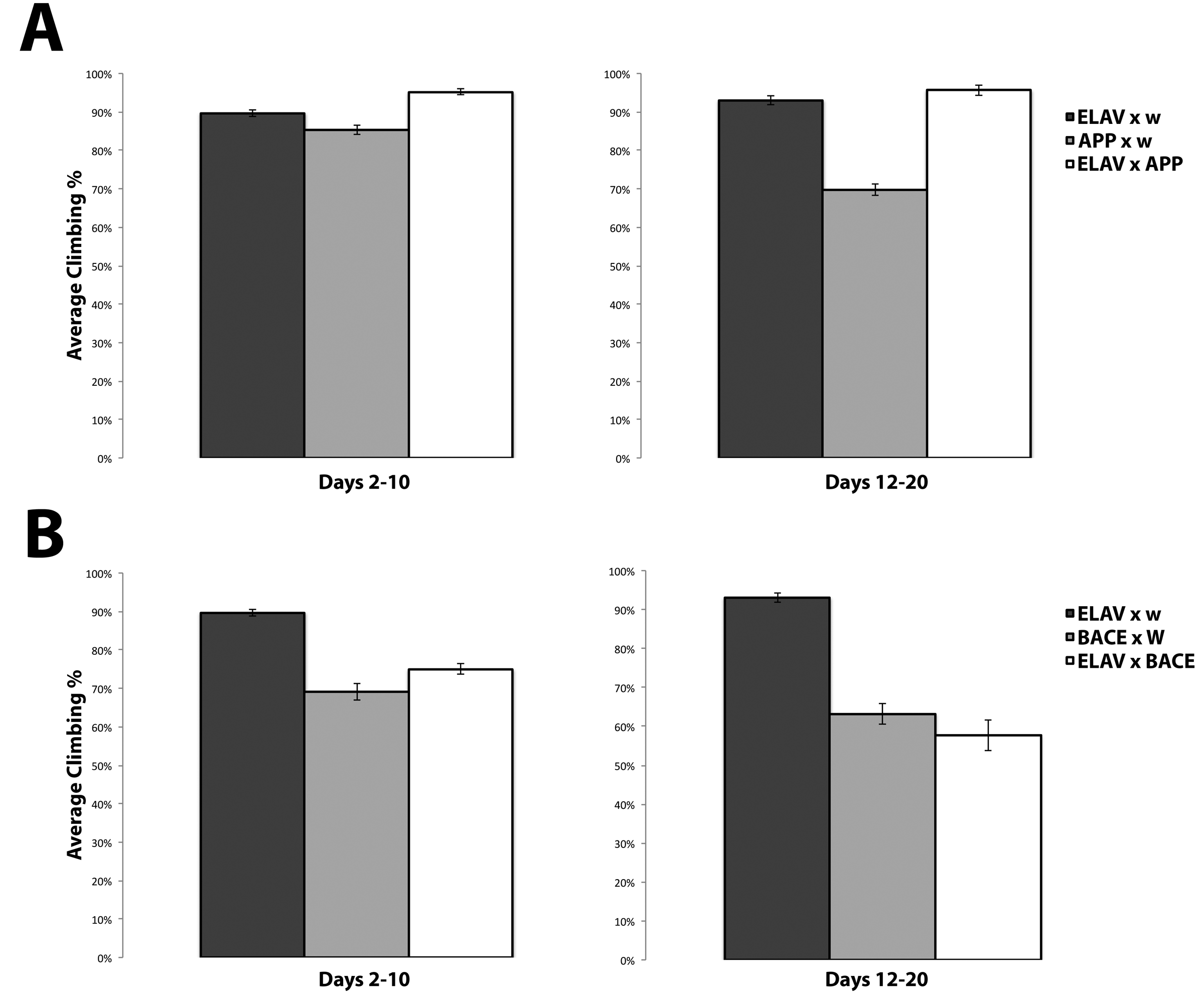

Supplement: Figure S4 — Motor reflex behavior. A) Expression of human APP alone does not change motor reflex behavior, as measured by the climbing assay. Parental strains listed. Error bars show standard error. No significant difference exists between elav; APP; + heterozygous flies and elav; +; + heterozygous flies in days 2–10 (ANOVA, p = .601) or in days 12–20 (p = .677). B) Expression of human BACE alone does not change motor reflex behavior (elav; BACE; + heterozygous flies). No significant difference was found between elav; BACE; + heterozygous flies and w; BACE; + heterozygous flies in days 2–10 (ANOVA, p = .106) or in days 12–20 (p = .066). Error bars represent standard error. (TIF) [file pone.0020799.s004.tif]
